# The development of malaria diagnostic techniques: a review of the approaches with focus on dielectrophoretic and magnetophoretic methods

**DOI:** 10.1186/s12936-016-1400-9

**Published:** 2016-07-12

**Authors:** Surasak Kasetsirikul, Jirayut Buranapong, Werayut Srituravanich, Morakot Kaewthamasorn, Alongkorn Pimpin

**Affiliations:** Department of Mechanical Engineering, Faculty of Engineering, Chulalongkorn University, Bangkok, 10330 Thailand; Animal Vector-Borne Diseases Research Group, The Veterinary Parasitology Unit, Department of Pathology, Faculty of Veterinary Science, Chulalongkorn University, Bangkok, 10330 Thailand

**Keywords:** Malaria, Dielectrophoresis, Magnetophoresis, Cell deformability, Cell morphology, Detection, Separation

## Abstract

The large number of deaths caused by malaria each year has increased interest in the development of effective malaria diagnoses. At the early-stage of infection, patients show non-specific symptoms or are asymptomatic, which makes it difficult for clinical diagnosis, especially in non-endemic areas. Alternative diagnostic methods that are timely and effective are required to identify infections, particularly in field settings. This article reviews conventional malaria diagnostic methods together with recently developed techniques for both malaria detection and infected erythrocyte separation. Although many alternative techniques have recently been proposed and studied, dielectrophoretic and magnetophoretic approaches are among the promising new techniques due to their high specificity for malaria parasite-infected red blood cells. The two approaches are discussed in detail, including their principles, types, applications and limitations. In addition, other recently developed techniques, such as cell deformability and morphology, are also overviewed in this article.

## Background

Malaria is a mosquito borne disease caused by protozoan parasites of the genus *Plasmodium*, and five species are reported for their infections in humans; namely, *Plasmodium falciparum, Plasmodium vivax, Plasmodium malariae, Plasmodium ovale* and *Plasmodium knowlesi* [1, 2]. Infection with different *Plasmodium* species, or sometimes with multiple species, results in different clinical outcomes in patients. The most virulent and fatal species of malaria is *P. falciparum*, especially when the infection occurs in young children with insufficient protective immunity, and in pregnant women. Although the other *Plasmodium* species might also cause severe illness in humans, the mortality rate is relatively low.

The 2014 World Health Organization report stated that there were about 584,000 malaria deaths annually worldwide, with 78 % of these deaths occurring in children under 5 years old [2], largely (>90 %) in Sub-Saharan Africa [2, 3]. In Thailand, the *Plasmodium* species that cause the majority of malaria in humans are *P. falciparum* (44 %) and *P. vivax* (47 %), and most cases are reported in regions bordering between Thailand and Myanmar [2–6]. In addition to *P. falciparum* and *P. vivax*, another malaria parasite species, *P. knowlesi*, is emerging as a health issue in Southeast Asia. This zoonotic species possesses the potential for infection of humans, with a natural reservoir in monkeys [7]. In some areas, for instance in Borneo hospitals of Malaysia, the prevalence of malaria infections by this parasite was as high as 83 % [8].

### Malaria life-cycle

Human-infecting *Plasmodium* relies on two hosts: humans and certain species of female *Anopheles* mosquitoes. When an infected mosquito bites a human, sporozoites from the salivary glands of the infected mosquito are injected into the bloodstream and enter the liver of the human, to initiate the exoerythrocytic stage of infection. Within the liver hepatocyte cells, the sporozoites transform and multiply via asexual reproduction to develop into schizonts, structures that can contain thousands of daughter parasites or “merozoites” [9].

After 6–8 days of development, depending on the *Plasmodium* species, fully mature schizonts rupture their host hepatocytes to release merozoites into the bloodstream [10]. The merozoites then invade red blood cells (RBCs) and undergo asexual development within the infected RBC (iRBC), referred to as the erythrocytic cycle. The erythrocytic cycle begins with a tiny ring form, followed by development to a larger amoeboid form termed the trophozoite and finally replication to merozoites within a mature schizont. After rupturing the host’s RBCs, the merozoites invade new RBCs, and the resulting cycles of amplification can result in severe pathology and suffering of the patient due to the burden of iRBC, such as anemia and sequestration of iRBC in deep tissues. Some of the merozoites will develop into male and female gametocytes following RBC invasion, which circulate in the blood and can be ingested by a female mosquito during a blood meal on the infected human. Fertilization between male and female gametocytes occurs in the mosquito midgut to produce zygotes. The zygotes further develop into ookinetes and traverse the mosquito midgut wall to transform into oocysts. Within the oocysts, sporozoites multiply, and the rupture of the mature oocysts release sporozoites that recognize and invade the mosquito salivary glands, where they are ready to continue the life cycle. The development of the malaria parasites in the *Anopheles* mosquito vector is termed the sporogonic cycle.

After merozoite invasion, the iRBCs undergo parasite-mediated structural transformations, such as remodeling of the iRBC membrane skeleton via exported parasite-encoded proteins. These physical changes include increased rigidity of the iRBC membrane, reduced iRBC deformability and an increased adhesiveness of the iRBCs [11, 12]. However, it was recently reported that there was an increased deformability of *P. vivax*-iRBCs compared to uninfected RBCs (hRBC), which is in contrast to that for *P. falciparum*-iRBCs [13]. Furthermore, other physical changes are due to one parasite-specific structure, the haemozoin or malaria pigment. This structure is produced from the haem groups released from the digestion of the haemoglobin within the parasite food vacuole, where the haem groups aggregate into an insoluble crystal [14].

Due to the difficulty in either detecting malaria-parasite infections, especially in a field setting, or separating infected erythrocytes for a biological study [15–17], many techniques have been developed to meet the specific requirements for each situation. This article aims to review recently developed techniques of malaria diagnosis, both for conventional techniques such as microscopy; rapid diagnostic test (RDT); molecular diagnoses, dominated by PCR-based techniques; and the alternative techniques of dielectrophoretic and magnetophoretic principles, which are based on physical properties of the iRBCs. In particular, the change in the dielectric and magnetic properties of the iRBC due to the released haemozoin has recently been considered as a key bio-marker for malaria diagnosis. Since the importance but difficulty of a correct diagnosis, techniques exploiting other physical and biological properties of iRBCs have also been developed, and some of these will be mentioned in this article.

## Conventional techniques

Microscopic diagnosis using blood smears plays an important role in malaria diagnosis because of its ability to diagnose and differentiate each species of malaria, and so it is used as the gold standard for any new detection tool or technique [see, for example, 18–22]. However, this method still suffers from drawbacks, such as requiring a visual or light microscope with 1000× magnification and relying on skillful and well-trained microscopists. Microscopic diagnosis is a morphology based identification so that *Plasmodium* species with closely similar in shape or characteristics such as *P. knowlesi* and *P. malariae* is prone to fault diagnosis, even by an expert. Recently, some researchers have introduced an image processing technique for microscopy, to avoid human error. However, the main cause of error that is due to a low parasite density was not resolved by this approach [23–25]. This is due to the fact that the average ability of microscopic diagnosis to detect *Plasmodium* in iRBCs has a threshold of around 10 parasites/µL for a research setting [26] and in the range of 50–100 parasites/µL for outside a research setting [27], or less sensitive in a limited resource setting. Fluctuations of parasite density over the course of infection contribute to detection-limit of microscopy-based diagnosis and all other direct detection approaches [28]. Consequently, direct detection at a single time point has likely resulted in under-estimation of malaria parasite infection rates, especially cases with a low parasite density and asymptomatic malaria at the early and chronic stage of infection. For example, Okell et al. reported that submicroscopic parasite carriage is commonly seen in adult patients in low-endemic settings [29].

Not only is microscopy laborious and ill-suited for high-throughput use, but in remote rural areas, such as peripheral medical clinics with no electricity and no health-facility resources, microscopy and skillful microscopists are often unavailable. Fortunately, this lack of microscopy in a rural area might be resolved in this near future due to the invention of an origami-based paper microscope [30]. This device was intentionally developed for malaria control in a poor country.

Another technique, the rapid diagnostic test (RDT), is likely to impact malaria control in the immediate future. A test-kit is able to immunologically detect a number of different malaria antigens such as lactate dehydrogenase (LDH), aldolase and histidine-rich protein-2 (HRP-2) in a small amount of blood (typically 5–15 μL) by a principle similar to that for strip pregnancy test. The detection is performed by an immunochromatographic assay with monoclonal antibodies directed against the target parasite antigen(s) that are impregnated on a test strip. The RDT strip has been extensively tested for its impacts on malaria diagnosis outside research settings for the past few years [5, 20–22, 31–35], where the implementation of RDTs increased the proportion of patients with a parasite-based diagnosis of malaria compared to microscopy alone, leading to a higher accuracy and timely clinical case management, as well as better cost effectiveness [33–35]. Over 200 RDTs have been currently tested and deployed in the field settings, the most popular ones are, for example, Binax NOW^®^, Optimal-IT^®^, Paracheck-Pf^®^ and Paramax-3 [36]. Some RDTs can detect only single species (either *P. falciparum* or *P. vivax*) while others detect multiple species (*P. falciparum, P. vivax, P. malariae* and *P. ovale*). Additionally, some RDTs further differentiate between *P. falciparum* and non-*P. falciparum* infection, or between specific species. No RDT specifically detects *P. knowlesi*, although Foster et al. reported that BinaxNOW^®^ correctly detected non-*P. falciparum* malaria in *P. knowlesi* samples but was the least sensitive, detecting only 29 % (8/28; 95 % CI 12–46 %) of fresh samples [37]. Although this technique is timely and easy to use, it is relatively expensive and prone to false-positive responses due to the persistence of malaria antigens in the blood for up to 2 weeks after the parasite has been cleared from the patient’s circulation. In addition, limits of detection (LOD) of these RDTs rely on an amount of antigen equivalent to 200 iRBCs/µL or 2000–5000 parasites/µL of blood [36].

The most efficient technique is PCR-based diagnosis which has produced a higher specificity and sensitivity in the identification and differentiation of malaria at the species level. PCR methods can be subdivided into nested PCR, semi-nested PCR, single step multiplex PCR, and real-time or quantitative PCR assays [38, 39]. Among them, the simplest and least technically demanding is a loop-mediated isothermal amplification (LAMP) assay [40]. Generally, PCR is able to detect parasites at a low density, typically below 5 parasites/μL of blood for all five human infecting *Plasmodium* parasites [40, 41]. Recently, it has been reported that saliva, urine and fecal samples of *P. falciparum*- and *P. vivax*-infected patients contain malarial DNA that is amplifiable by PCR [42, 43]. Although PCR-based diagnosis has a high sensitivity and good specificity, it relies on the complimentary nucleotide sequences between the primer (known sequence) and its counterpart target DNA (unknown sequence), and so parasites with genetically diverse sequences at primer’s target region are prone to detection failure or to a lower amplification efficiency that will reduce the sensitivity of the PCR test. In addition, the sensitivity of detection by PCR-based techniques is affected greatly by copy number of target gene or nucleic acid sequence available in the *Plasmodium* genome. The most widely used target is small subunit 18S ribosomal RNA gene (18S rDNA). This gene is highly conserved across *Plasmodium* species and has a moderate copy number-presenting in four to eight copies per parasite. Mitochondrial DNA is another promising target due to it its greater abundance than nuclear DNA (between 30 and 100 copies per parasite). Over the past decade, research efforts have increasingly utilized a well conserved cytochrome b gene in mitochondria genome not only in human *Plasmodium* [44], but recently also proven successful in microscopically negative samples in a newly rediscovered *Plasmodium* species in ungulates [45]. More PCR-based approaches including wide variety of target DNA or RNA transcripts and their limitations can be seen in recent reviews including Refs [10, 29, 46].

Table 1 shows a comparison of the performance for each diagnostic technique. Currently, PCR has the lowest detection range of the parasite (around 1–5 parasites/µL), but the diagnostic cost is still high, and accessibility is restrictive.

**Table 1:** Comparison of performance between different malaria-diagnosis techniques

Technique	Detectable parasite density (per µL)	*Plasmodium* species	Infected stage	Sensitivity (%)	Operation cost (per test)	Parasite enrichment	Heterogeneity		Operation time (per test)
							Stage dependence	Cell-size dependence	
Microscopy	5–20 [47], 50–100 [27]	All species (depending on expertise)	All stages (depending on expertise)	Gold standard	$5000 for a microscope $0.12–$0.40 for a test [18]	No	No	No	30–60 min [41]
RDTs									
HRP-2	>100 [48]	*P. falciparum*	No data available	96.9 % [48]	$0.55–$1.50 [18]	No	No	No	~20 min [41]
pLDH	>100 [48]	All species	No data available	91.2 % [48]	$0.55–$1.50 [18]	No	No	No	~20 min [41]
PCR	1–5 [19]	All species (depending on the primer set)	All stages	~100 % when density >5000/µL for *P. falciparum* and 500/µL for *P. vivax* [4]	$100 for a PCR thermal cycler $3.3 for a test [49]	No	No	No	~24 h [41]
DEP deformation [50]	No data available	*P. falciparum*	All stages	No data available	<0.1$ per test (approx.)	Yes	No data available	Yes	Depending on volume of sample
Electromagnet with wedge-shaped pole [51]	No data available	*P. vivax* *P. malariae*	No data available	*20* *% for P. malariae* *25* *% for P. vivax*	<0.1$ per test (approx.)	Yes	Yes	Yes	6–12 h [51]
Magnetophoretic between stainless wool in a large chamber [52]	5000 for 1st chamber and 50 for 2nd chamber (10^7^ and 10^5^/2 mL)	*P. falciparum*	Trophozoites and schizonts	No data available	<0.1$ per test (approx.)	Yes	Yes	Yes	~15 min [52]
Magnetophoretic in a microchannel between two magnets [53]	No data available	*P. falciparum* *P. vivax* *P. malariae* *P. ovale*	Trophozoites, schizonts, gametocyte	No data available	<0.1$ per test (approx.)	Yes	Yes	Yes	Depending on volume of sample
Magnetophoretic between stainless wool in a commercial column tube [54]	400 (10^7^ per 25 mL)	*P. falciparum*	Trophozoites and schizonts	95.7 %	<0.1$ per test (approx.)	Yes	Yes	Yes	Depending on volume of sample
Magnetophoretic with magnet nanoparticles [55]	30	No data available	No data available	No data available	<0.1$ per test (approx.)	Yes	No data available	No data available	Depending on volume of sample
Magnetophoretic with ferromagnetic material [56]	No data available	*P. falciparum*	All stages	99.2 % for late-stage iRBCs, 73 % for ring-stage iRBCs at optimal flow (0.14 μL/min)	<0.1$ per test (approx.)	Yes	Yes	Yes	Depending on volume of sample
Magnetic relaxometry detection [57, 58]	<10 [58]	*P. falciparum*	All stages	~100 %	No	Yes	Yes	~30 min	

Remark: sensitivity is the probability of a positive test result for a patient with the disease

## Dielectrophoretic principle

**Fig. 1:**
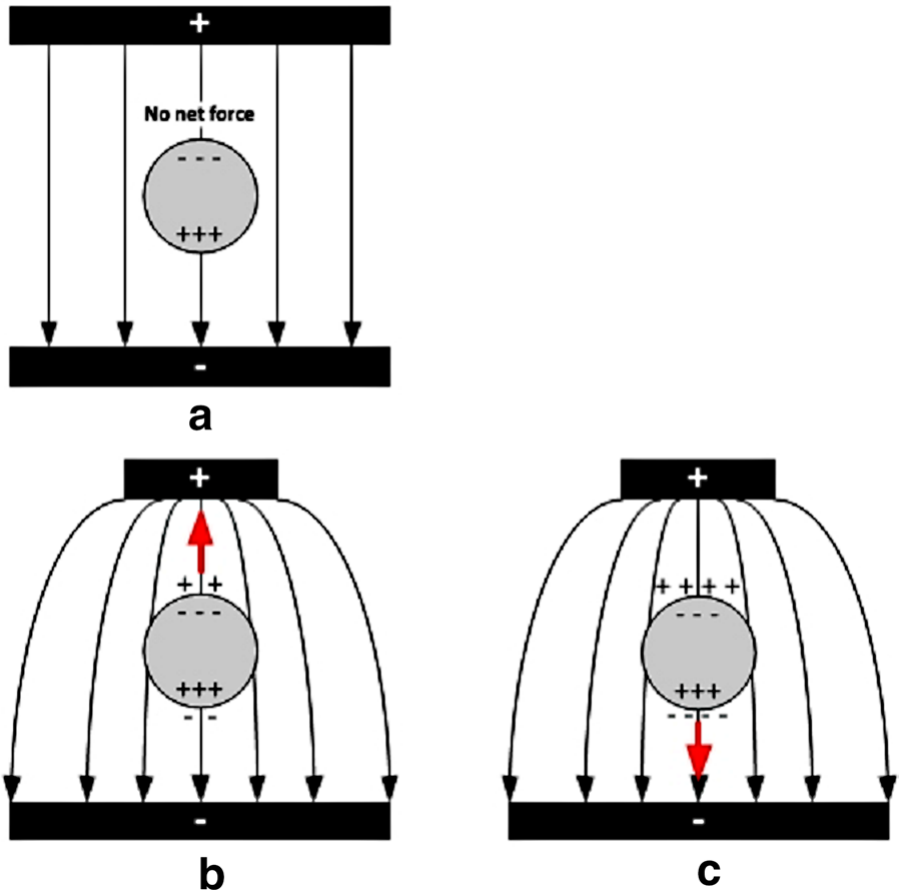
Comparison of the net force between different situations: **a** uniform electric field, **b** positive dielectrophoresis and **c** negative dielectrophoresis

Dielectrophoresis (DEP) was first introduced in 1951 by Pohl [59]. It is a phenomenon in which polarized neutral particles exposed to a non-uniform electric field are pushed towards or against high electric field intensity regions. The principle of DEP is illustrated in Fig. 1a–c. A neutral particle exposed to an electric field experiences polarization that causes positive and negative charge accumulation at the opposite sides of the particle. In a uniform electric field, the electrostatic forces are equal on each side of the particle so there is no net force (Fig. 1a). In contrast, the electrostatic forces are unequal when the particle is exposed to a non-uniform electric field due to the heterogeneous electric field strength experienced across the particle (Fig. 1b, c). Therefore, the particle experiences a net dielectrophoretic force, which causes particle motion. The direction of the force can be explained in terms of charge accumulation at the interfaces of the particle. In Fig. 1b, in which a particle is more polarizable than the suspension medium, the dielectrophoretic force drives the particle towards the high electric field intensity region, known as a positive dielectrophoresis (pDEP). When the particle is less polarizable than the medium, the dielectrophoretic force pushes the particle towards the low electric field intensity region (Fig. 1c), known as a negative dielectrophoresis (nDEP).

**Fig. 2:**
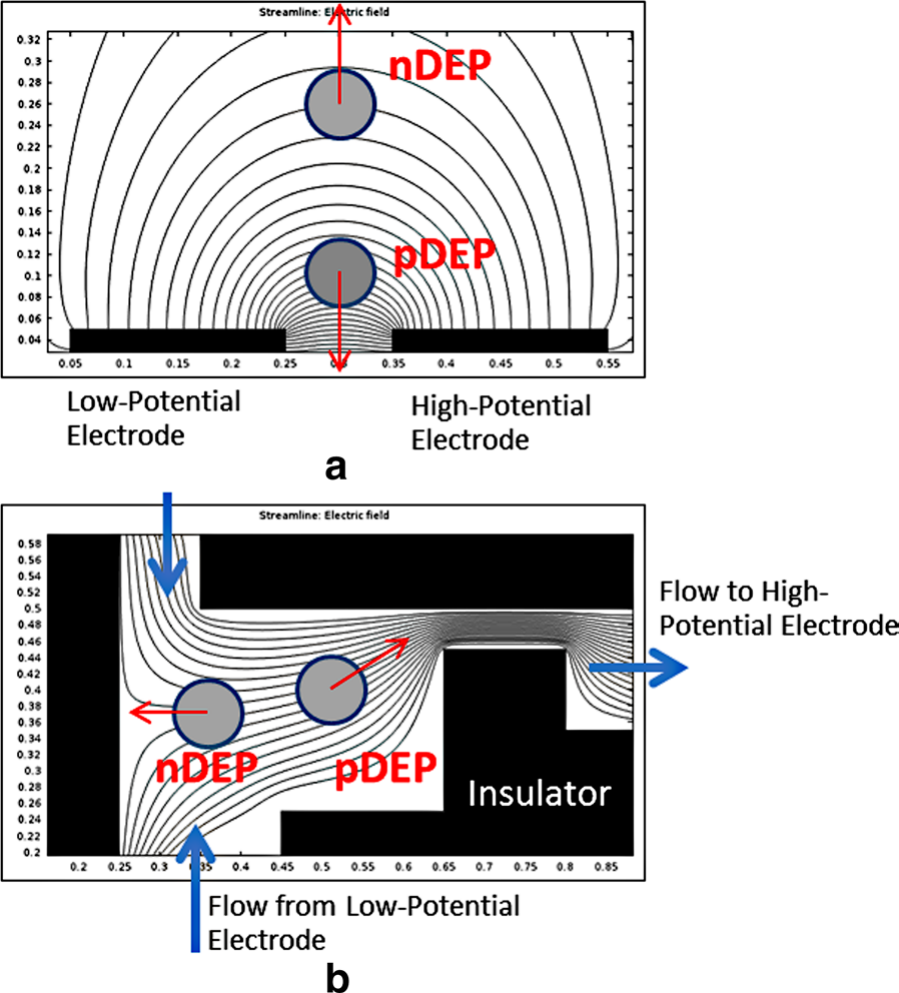
Two methodologies to create a non-uniform electric field: **a** asymmetric electrode pair and **b** asymmetric microchannel

At present, DEP has been performed with alternating current (AC), direct current (DC), combined AC/DC and travelling wave, but AC-DEP is the classic DEP that has been used in diverse applications [60–68]. The electrodes used to generate the non-uniform electric field are located inside a microchannel in which the samples are fed (Fig. 2a). The merit of AC-DEP over the other DEP techniques is its flexibility to manipulate various types of particles due to their frequency-dependent dielectric properties. The direction of the force (pDEP or nDEP), as well as the magnitude, can be controlled by adjusting the signal frequency and electric potential. However, there are a few drawbacks, such as electrode fouling and bubble occurrence inside the channel due to the electrolysis of suspension medium during its operation.

Also known as insulator-based DEP (iDEP), DC-DEP utilizes DC applied to electrodes to generate a non-uniform electric field [69–71]. Typically, the electrodes would be located outside a flow channel, while nonconductive insulators are fabricated as a channel wall to create electric-field non-uniformity in a flow passage (Fig. 2b). This technique helps eliminate the fouling of electrodes as well as the bubbles inside the channel, since there is no contact between the sample medium and electrodes. The main drawback of the DC-DEP approach is the required use of a relatively higher electric potential that might cause a Joule heating problem. The combination of AC/DC-DEP would eliminate the drawbacks of the DC-DEP technique by reducing the electric potential required as well as the Joule heating problem [72–74].

Travelling wave DEP utilizes only AC to manipulate and transport particles [75, 76]. Although the mechanism of particle manipulation is similar to that for AC-DEP, there are phase shifts amid the generated electric fields that help serially carrying the particles between each electrode pair.

Although the AC-DEP technique has a few drawbacks, it is still the most practical method for this application. Accordingly, this section will describe the fundamentals of AC-DEP to illustrate the key concepts. The simplified model to derive the time-average AC-DEP expression for a spherical particle is based on the dipole approximation that is given by Eq. (1) [77];

1 $$\overset{\lower0.5em\hbox{$\smash{\scriptscriptstyle\rightharpoonup}$}} {F}_{DEP} = 2\pi R^{3} \varepsilon_{m} Re[f_{CM} ] \nabla \left| {E_{rms} } \right|^{2} ,$$

where *R* is the particle radius, *ɛ*_*m*_ is the dielectric permittivity of the medium, *Re*[*f*_*CM*_] is the real value of the Clausius–Mossotti factor and *E*_*rms*_ is the root mean square of the electric field intensity. The complex Clausius–Mossotti factor is defined by Eq. (2);

2 $$[f_{CM} ] = \left\{ {\frac{{\left( {\varepsilon_{p} + {{\sigma_{p} } \mathord{\left/ {\vphantom {{\sigma_{p} } {j\omega }}} \right.} {j\omega }}} \right){ - }\left( {\varepsilon_{m} + {{\sigma_{m} } \mathord{\left/ {\vphantom {{\sigma_{m} } {j\omega }}} \right.} {j\omega }}} \right)}}{{\left( {\varepsilon_{p} + {{\sigma_{p} } \mathord{\left/ {\vphantom {{\sigma_{p} } {j\omega }}} \right.} {j\omega }}} \right) + \text{2}\left( {\varepsilon_{m} + {{\sigma_{m} } \mathord{\left/ {\vphantom {{\sigma_{m} } {j\omega }}} \right.} {j\omega }}} \right)}}} \right\} ,$$

where *ɛ*_*p*_ and *σ*_*p*_ are the dielectric permittivity and electrical conductivity of the particle, *ɛ*_*m*_ and *σ*_*m*_ are the dielectric permittivity and electrical conductivity of the medium, and *ω* is the signal frequency. The AC-DEP equation analogous to Eq. (1) for a multi-pole model has been derived elsewhere. The direction of the force exerting on a particle relies on the real value of the complex Clausius–Mossotti factor (*Re*[*f*_*CM*_]), which is related to the effective polarizability. The factor depends upon the electrical conductivity and the dielectric permittivity of the particles and the suspension medium, as well as the applied signal frequency. It should be noted that Eq. (2) can be employed not only for the single-shell model but also for the multi-shell model. However, further modifications for the multi-shell approximation are needed, such as described in Ref. [77].

In general, it should be understood that each type of biological cell, as well as particle, has its own frequency-dependent characteristic leading to a different magnitude and direction of exerted DEP force in a non-uniform electric field. Therefore, AC-DEP-based micro-devices have been applied for biomedical applications to separate, sort, trap and focus biological cells, such as cancer cells, stem cells and infected cells [60–62], in addition to bacteria, viruses, DNA and proteins [63–68].

With respect to the application for malaria parasite detection, Gascoyne et al. studied the changes in the dielectric properties of iRBCs compared to hRBCs using electrorotation (ROT) and dielectrophoretic crossover approaches [78]. They found that the dielectrophoretic crossover method was better able to predict the dielectric properties of iRBCs than the ROT approach. A summary of their experimental results [62] for a spherical shell model is shown in Table 2. From these results, they explained that the membrane conductivity of iRBCs was higher than that of hRBCs because of RBC membrane barrier deterioration during malaria parasite infection. However, iRBCs retain their internal ions, as shown by their relatively high internal conductivity.

**Table 2:** Dielectric properties of iRBC and hRBC [62]

Cell type	Position	Electrical conductivity (S/m)		Relative dielectric permittivity	
		Host	Parasite	Host	Parasite
iRBC	Membrane	7 ± 2 × 10^−5^	<10^−6^	9.03 ± 0.82	8 ± 4
	Interior	(0.95 ± 0.05)σ_m_	1.0 ± 0.4	58 ± 10	70 ± 5
hRBC	Membrane	<10^−6^		4.44 ± 0.45	
	Interior	0.31 ± 0.03		59 ± 6	

Remark: σ_m_ is the electrical conductivity of the suspension medium

Subsequently, iRBCs were isolated away from hRBCs using two types of electrode configuration (interdigitated and spiral electrodes) [62]. The system was designed in a manner that the iRBCs experienced a relatively weak nDEP while hRBCs were manipulated under a relatively strong pDEP. The interdigitated electrode was energized by a single-phase sinusoidal signal up to 5 V_p-p_ at a frequency range from 1 to 5 MHz, resulting in the hRBCs being attracted to the electrode edges, while the iRBCs were pushed to the electrode gaps. As a result, iRBCs were swept away by the hydrodynamic force of the suspension medium resulting in a 200-fold concentration of the iRBCs. The other electrode design employed a spiral array of four parallel electrodes. The electrode was activated by a quadrature-phase sinusoidal signal up to 5 V_p-p_ in the frequency range from 1 to 15 MHz, while the second electrode created both traditional AC-DEP and travelling wave. After applying the electric signal, hRBCs were trapped at the electrode edges while iRBCs were levitated and carried towards the center of the electrode, resulting in an approximate 1000-fold enrichment of iRBCs.

**Fig. 3:**
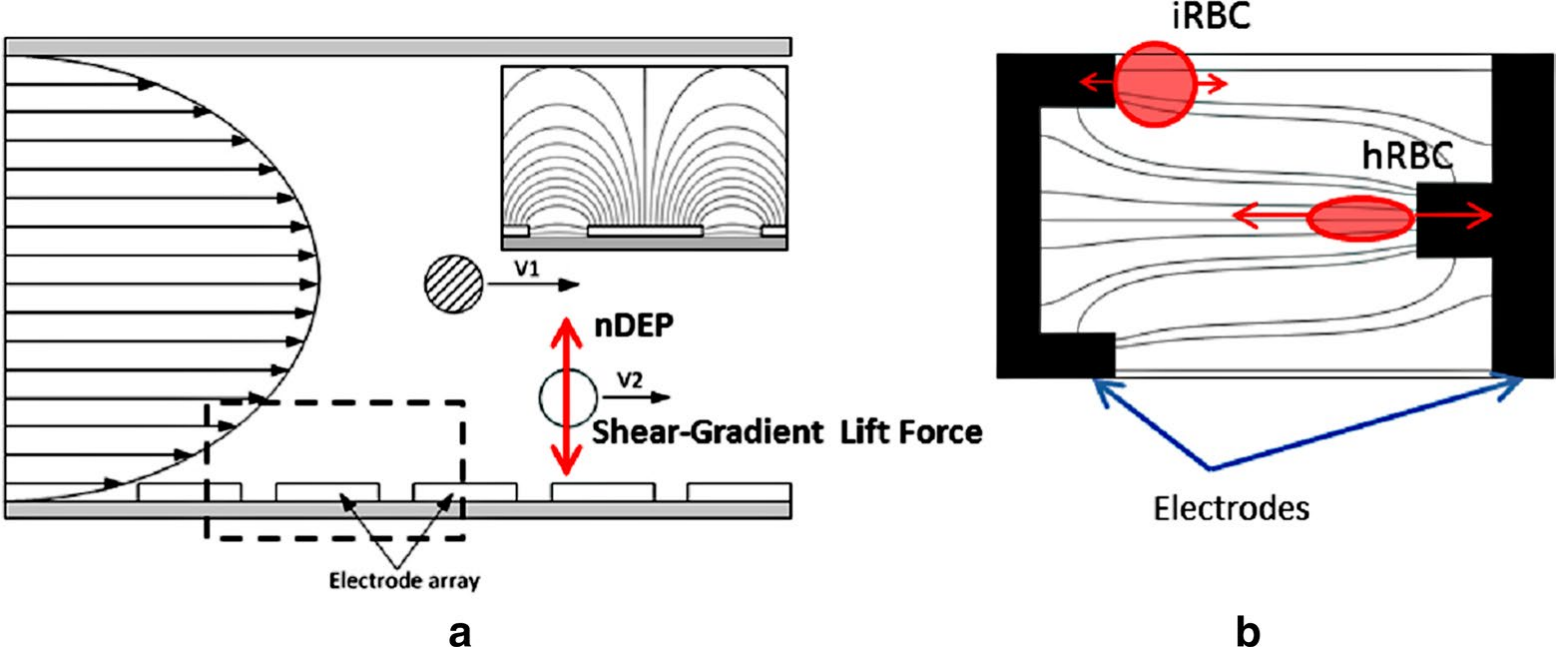
Two applications of the dielectrophoretic force: **a** cell separation and **b** cell characterization

In 2004, Gascoyne et al. [79] proposed a new approach to diagnose malaria, relying on an automated micro-total analysis system (μTAS). The so-called DEP-field-flow-fractionation (DEP-FFF) approach was employed to isolate iRBCs from hRBCs. Simplistically, DEP-FFF is another version of DEP that uses the dielectrophoretic force to position particles in a fluid stream. Different types of particles experience a distinct velocity in accordance with their respective positions due to the parabolic velocity profile of the flow stream (Fig. 3a). The discrepancy in their velocity causes the particles to emerge at the end of the channel at different times. In the study of Gascoyne et al. [79], electrode arrays on the channel wall were applied by sinusoidal signals up to 5 V_p-p_ in the frequency range from 40 to 250 kHz and the successful separation of iRBCs from hRBCs by the DEP-FFF approach was obtained. However, the test of integrated flow-through PCR for malaria diagnosis was not included in that report.

Recently, AC-DEP was used not only to discriminate early stage iRBCs from hRBCs but also used to predict cellular mechanical properties [50]. In this study, both iRBCs and hRBCs were trapped at the electrode edges when the electrodes were activated. The iRBCs were able to retain their original shape at the edges due to their greater stiffness and weaker DEP force. In contrast, the hRBCs were significantly deformed due to their stronger pDEP effect. The noticeable deformation of hRBCs over iRBCs offered the possibility of a visible and mechanical discrimination (Fig. 3b). In addition, the cell deformability could be measured by estimating the DEP force exerted on the hRBCs from Eq. (1) and the stretch ratio.

The AC-DEP method exploits dissimilar dielectric properties between iRBCs and hRBCs for cell discrimination and might be the most appropriate of the different DEP techniques for malaria parasite detection. However, due to the low difference in, and the heterogeneity of, the electrical properties and diameter of iRBCs and hRBCs, the employment of the dielectrophoretic approach is quite limited in malaria parasite research.

## Magnetophoretic principle

With respect to the life cycle of the malaria parasite, after each sporozoite invades a liver cell and undergoes asexual development followed by hepatocyte lysis, the resulting merozoites are swept into the bloodstream to infect RBCs. The parasite digests haemoglobin leaving haem groups that aggregate into an insoluble brown pigment haemozoin. The number and size of haemozoin pigments depends on the developmental stage of the parasite [80, 81]. Accordingly, the level of haemozoin in RBCs has become a key physical property that is used to detect or separate malaria parasite infected RBCs. Studies using spectroscopic and crystallographic techniques revealed that haemozoin has a magnetic structure that is a synthetic biomineral, specifically, beta-haematin [82]. The molecular structure and composition of beta-haematin is generally a single domain crystal of magnetite (Fe_3_O_4_) or greigie (Fe_3_S_4_) [83, 84]. Additionally, its magnetic properties have been determined using electron paramagnetic resonance and Moessbauer spectroscopy, and the results revealed that haemozoin has a Fe^3+^ component [85]. The magnetic properties of iRBCs were quantified, where the magnetophoretic mobility and net volume magnetic susceptibility of iRBCs were found to be 2.94 × 10^−6^ mm^3^ s/kg and 1.80 × 10^−6^ (relative to water), respectively, at a haemoglobin to haemozoin fraction of 0.5 [80, 86].

When a magnetophoretic force acts on a particle that lies in a non-uniform magnetic field, the magnitude of the magnetophoretic force is given by Eq. (3);

3 $$F_{m} = 2\pi \mu_{medium} a^{3} \left( {\frac{{\mu_{particle} - \mu_{medium} }}{{\mu_{particle} + 2\mu_{medium} }}} \right)\nabla \left| {\vec{H}} \right|^{2} ,$$

where *μ*_*medium*_ is the magnetic permeability of the suspension medium (N/A^2^), *μ*_*particle*_ is the magnetic permeability of the particles (N/A^2^), *a* is the radius of the particles and *H* is the magnetic field strength (A/m). Considering Eq. (3), the magnitude of magnetophoretic force is proportional to the cubic size of the particles and also the gradient of magnetic field. Thus, Eq. (3) can be simplified to that of Eq. (4);

4 $$F_{m} = \frac{2}{3}\pi \mu_{0} \Delta \chi a^{3} \nabla\left| {\vec{H}} \right|^{2} ,$$

where *µ*_*0*_ is the permeability of free space (4π × 10^−7^ N/A^2^) and $\Delta \chi$ is the relative magnetic susceptibility of particles to suspension medium (*χ*_*target*_ − *χ*_*buffer*_). From Eq. (4), the magnitude of the magnetophoretic force is proportional to the relative magnetic susceptibility. The magnetophoretic principle for malaria parasite detection then takes advantage of the significantly different magnetic properties between hRBCs and iRBCs, as shown in Table 3.

**Table 3:** Relative magnetic susceptibilities of each type of RBC to water [57]

Type of RBC	Relative magnetic susceptibilities ($\Delta \chi$) 10^−6^
hRBC	0.01
Early ring form-iRBC	0.82
Late trophozoite-iRBC	0.91
Schizont-iRBC	1.80

**Fig. 4:**
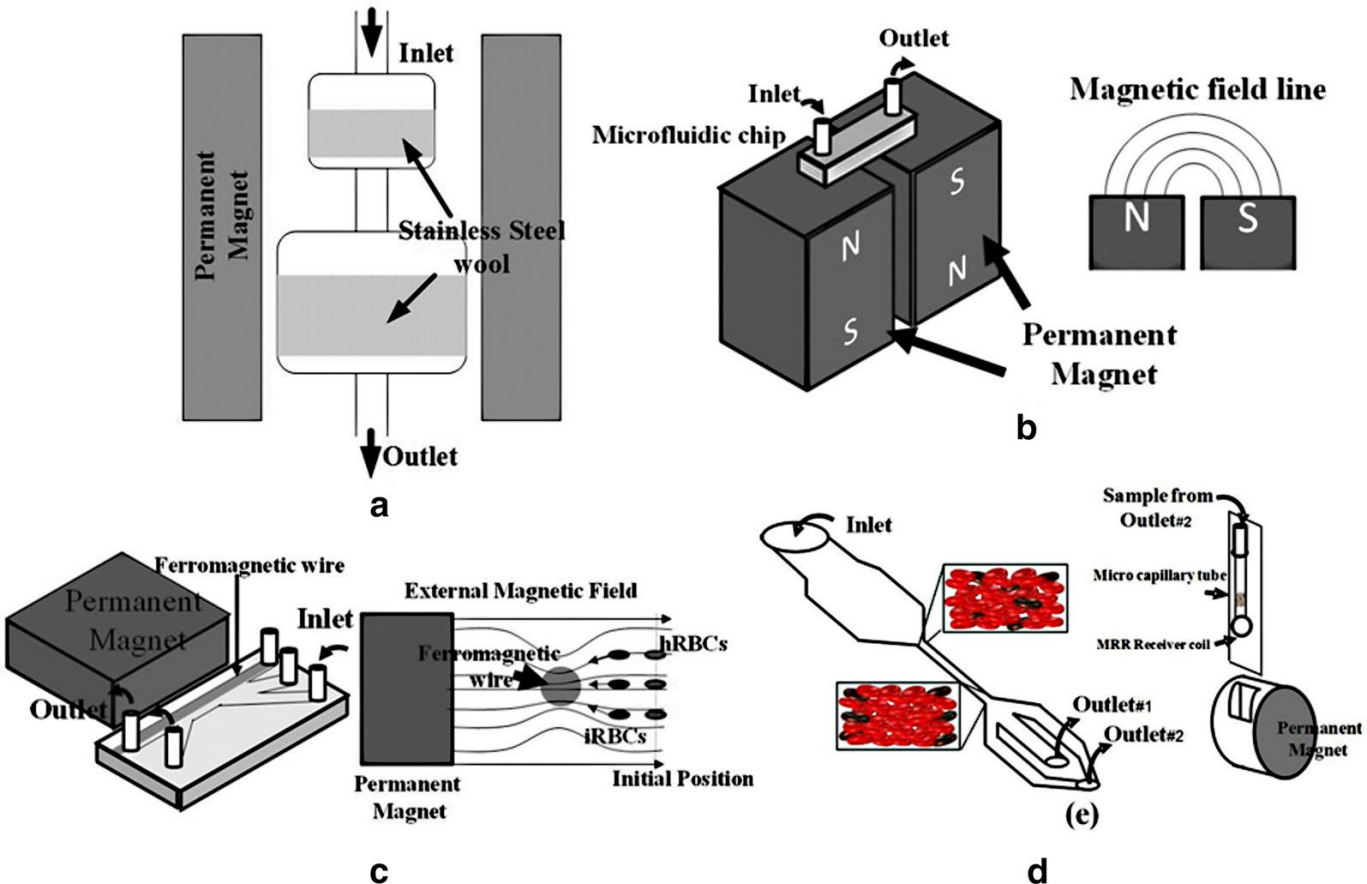
Applications of magnetophoresis: **a** locally strengthening the force using metal wool, **b** switching the magnetic field direction using opposing poles, **c** creating the non-homogenous field using a ferromagnetic wire and **d** manipulating cells to a location under high magnetic force using a centrifugal force

Paul et al. were probably the first research group to apply a magnetophoretic force for separating iRBCs in practice [52, 87], in which they used a fluid channel containing two chambers of dimensions of 25 × 10 × 52 mm^3^ (first chamber) and 13 × 13 × 9 mm^3^ (second chamber). Both chambers were filled with 25-µm diameter stainless wire at 4.6 and 7.6 % density for the first and second chamber, respectively. The channel was placed between a 0.7 T permanent magnet, as shown in Fig. 4a. They reported that about 75 % of iRBCs in the trophozoite and schizont stages were immobilized in the chamber under an operating flow velocity of 0.19 mm/s. A similar idea was later applied by employing a 0.7 T yoke-shaped neodymium magnet of 50 × 30 × 12 mm^3^ with a microchannel between the dipole magnets [54]. The channel contained stainless steel wool with a size range from 30 to 50 µm diameter and was flushed with isotonic sucrose solution containing 0.75 % (w/v) gelatin for the concentration stage and 0.2 % (v/v) RPMI 1640 and 1 % (w/v) BSA for the subsequent depletion stage. The device could increase the concentration of iRBCs up to 96 %, where most of the trapped iRBCs were in the schizont stage due to their high magnetic susceptibility. It should be noted here that some types of stainless steel wool could help inducing a local magnetic field gradient as well as magnetophoretic force, depending on their composition.

Subsequently, Zimmerman et al. [53] used two ferrite permanent magnets separated by 1.27 mm to trap iRBCs inside a microchannel. The magnetic field strength at the midline between the surfaces of the two permanent magnets was 1.1 × 10^6^ A/m. At this position, where the strongest magnetic field gradient was induced, iRBCs would be forced to move towards the gap between the two magnets perpendicularly to the mainstream of blood flow in the channel (Fig. 4b). At a velocity of 1.2 mm/s, the concentration of iRBCs was increased 40-, 250-, 250- and 375-fold for *P. falciparum*, *P. vivax*, *P. malariae* and *P. ovale*, respectively.

Another approach for malaria diagnosis is to utilize the changes in both the magnetic and optical properties of the iRBCs, since they exhibit a high magnetic anisotropy and have optical dichroism properties. Based on this principle, an iRBCs detection technique was developed using the magneto-optic principle, which allowed a high-sensitivity detection of the malaria pigment [88, 89].

An additional approach for the magnetophoretic principle is the employment of magnetic nanoparticles as label markers [55]. The addition of magnetic nanoparticles to attach a magnetic crystal of β-haematin, which can amplify the Raman spectroscopy signal, allowed the detection of iRBCs at an early stage. The other approach is to separate the iRBCs under a continuous flow in a microfluidics channel. For this system, an applied uniform 0.6 T magnetic field across a 100-µm diameter nickel wire along the channel (Fig. 4c) was reported [56]. The ferromagnetic wire increased the magnetic field gradient resulting in a more easily controlled lateral movement of the iRBCs away from the mainstream of blood flow. Overall, a separation efficiency of almost 100 % was obtained at a flow rate of <0.14 μL/min.

Recently, the magnetophoretic force has been combined with other physical properties, such as cell rigidity, to detect or separate iRBCs [57, 58]. A schematic illustration of one such device is shown in Fig. 4d. After the blood sample flows through a narrow channel of 50 μm width and 25 mm length, the eluted blood was centrifuged with an accumulation of iRBCs relatively close to the wall of the microchannel. The channel was then put in a micromagnetic resonance relaxometry (MRR) receiver coil with a 0.5  T permanent magnet, where the presence of iRBCs in the blood sample was detected and the signal transmitted through the MRR spectrometer.

These studies using the magnetophoretic principle employed an external magnetic field, where it was found that the detection or separation performance could be enhanced, but to a limited extent. This was due to the fact that the system could not provide a sufficiently high magnetic-force locally to effectively and precisely manipulate iRBCs. To resolve this issue, a system providing a high magnetic field gradient would be a key component in generating a high magnetophoretic force [90]. One promising technique is the addition of a ferromagnetic substance into the system. The substance can modify the magnetic field strength profile in the area around it. However, the gradient of magnetic field resulting from this ferromagnetism happens only in a tiny area around the ferromagnetic substance. Considering this factor, the employment of a microfluidics system would be an appropriate choice for malaria parasite detection. Furthermore, a high magnetic field gradient can be generated by various other factors, such as the type of ferromagnetic material, the orientation of the component and the direction of the uniform external magnetic field.

## Recent trends

From the literature, many studies take advantage of the existence of iRBC-specific haemozoin, which is a prominent biomarker for malaria diagnosis without sophisticated sample preparation. However, the detection or separation-based on haemozoin alone might have several pitfalls causing low efficiency of diagnosis. For example, electrical properties (see in Table 2) of uninfected and infected red blood cells are not significantly different; therefore, a separation of iRBCs with dielectrophoretic force alone may be impractical. Moreover, iRBCs at the ring stage contain low concentration or non-existent haem crystal [91]. These factors might have been responsible for the low efficiency haemozoin-based detection and separation of early stage infections in several past studies.

On the contrary, one of outstanding capabilities of dielectrophoresis and magnetophoresis (see in Table 1) is its ability to enrich the concentration of infected erythrocytes. The enrichment and separation without destroying of the parasite, which could not be obtained from microscopy, RDTs and PCR techniques, might be invaluable in malaria parasite research. Additionally, because of the portability, minimal expertise and ease in use, methods based upon dielectrophoretic and magnetophoretic principles could be considered as an alternative for malaria diagnosis.

In the last few years several research groups have tried to employ other new techniques to enhance the detection or separation of iRBCs. One of these approaches is the use of non-woven fabric (NWF) size-based filtration, which is simple and cheap [92]. The prototype single-use NWF filter demonstrated the successful removal of 99 % leukocytes from 5 mL of malaria-infected blood, which helped reducing the white blood cells contamination of iRBCs during a biological study. Another study found that the morphological changes on the surface of the malaria-iRBCs could be a biomarker for malaria diagnosis [93].

In addition to the physical change in RBCs following malaria infection, the reduced deformability of iRBCs is a property that could be employed for malaria detection and separation, at least for *P. falciparum*-iRBCs. Moreover, it has been reported that the iRBC deformability could be used to sort *P. falciparum*-iRBCs at different developmental stages and to enrich the ring-stage by 2500-fold [94]. Several other works have studied the relationship between the iRBC deformability and developmental stage of the infecting *Plasmodium* [95, 96] and the possibilities of a deformability-based technique to sort iRBCs [97–100].

More recent research reported that the high optical absorbance and nanosize of haemozoin could generate a transient vapor nanobubble around haemozoin when exposed to a short NIR picosecond laser pulse. The acoustic signals of these induced nanobubbles provided a transdermal, non-invasive and rapid detection of malaria infection in animals without using any reagents or drawing blood [101].

Several studies have employed a new platform to an existing conventional principle. For example, flow cytometry using standard nucleic acid-based staining methods was employed for a rapid and highly sensitive detection of iRBCs [102, 103], while a paper-based 2D platform that enabled multistep assays was also developed to detect malaria antigens in a low-resource setting [104]. A droplet microfluidics platform was employed for highly sensitive and quantitative detection of iRBCs based on an enzyme activity measurement [105]. In addition, a combination of dielectrophoretic and magnetophoretic forces has also been employed for the separation of iRBCs [106].

## Conclusions

Globally, around 3.2 billion people are estimated to be at risk of being infected and developing malaria, and 1.2 billion people are at a high risk. According to the latest estimation, over 200 million cases of malaria occur around the world annually, and the disease leads to over 500,000 deaths each year. Early detection could save lives and prevent disease outbreaks, as well as allow parasite enrichment for biology studies, which are important topics in malaria research. The conventional microscopic examination of blood smears, antigen-based rapid test and molecular biology-based diagnosis all have some limitations for effective employment in low-resource setting areas. Therefore, researchers around the world are looking for new options. Among various techniques, dielectrophoretic and magnetophoretic principles have recently became attractive possibilities for malaria diagnosis due to the unique changes in the electrical and magnetic properties of iRBCs compared to hRBCs. Moreover, alternative techniques, such as cell morphology and deformability, have been proposed to be suitable bio-markers for the future. Not only is a knowledge of parasite biological properties critical, but the development of an engineering system is also an important factor, and should be studied in parallel. One example is a magnetic-field generating system that could create a high magnetic field gradient over a large area. Another solution might be a combination of two or three detection techniques, which might be able to increase the specificity to iRBCs and so enhance the ability for malaria detection or infected erythrocyte separation.
